# Supplementary material for: Semi-supervised learning improves regulatory sequence prediction with unlabeled sequences
Source: BMC Bioinformatics. 2023 May 5;24:186. doi: 10.1186/s12859-023-05303-2 (PMC10163727; doi:10.1186/s12859-023-05303-2)
Supplement: Supplementary file 1 — Additional file 1: Fig S1. Scatterplots of observed CTCF ChIP-seq signal compared to predicted signal by semi-supervised model (here called CNN-GNN) and by the baseline model (CNN) where graph convolution was not used. [file 12859_2023_5303_MOESM1_ESM.pdf]

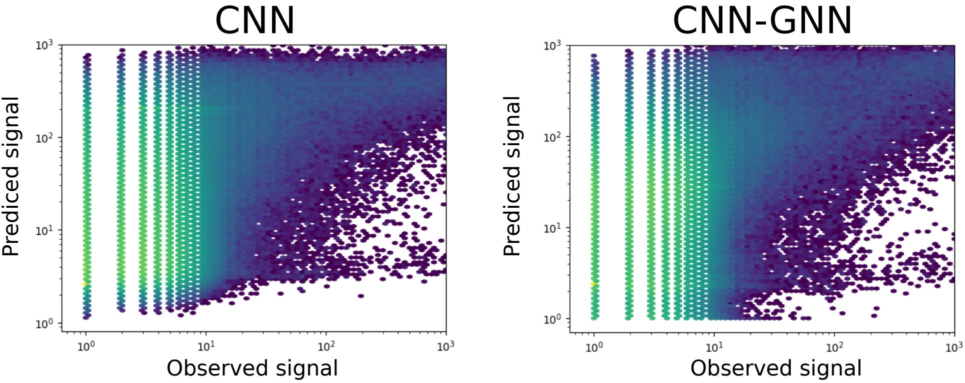

Supp Fig S1: Scatterplots of observed CTCF ChIP-seq signal compared to predicted signal by semi-supervised model (here called CNN-GNN) and by the baseline model (CNN) where graph convolution was not used.
