# Supplementary material for: Semi-supervised learning improves regulatory sequence prediction with unlabeled sequences
Source: BMC Bioinformatics. 2023 May 5;24:186. doi: 10.1186/s12859-023-05303-2 (PMC10163727; doi:10.1186/s12859-023-05303-2)
Supplement: Supplementary file 2 — Additional file 2: Fig S2. Comparison of prediction performances between the semi-supervised (here called CNN-GNN) with the baseline model (CNN) where graph convolution was not used for NANOG, OCT4 and SOX2 peak classifications. Ten trainings were done for each model to make boxplots. Area under roc curve (AUROC). Area under the precision recall curve (AUPR). [file 12859_2023_5303_MOESM2_ESM.pdf]

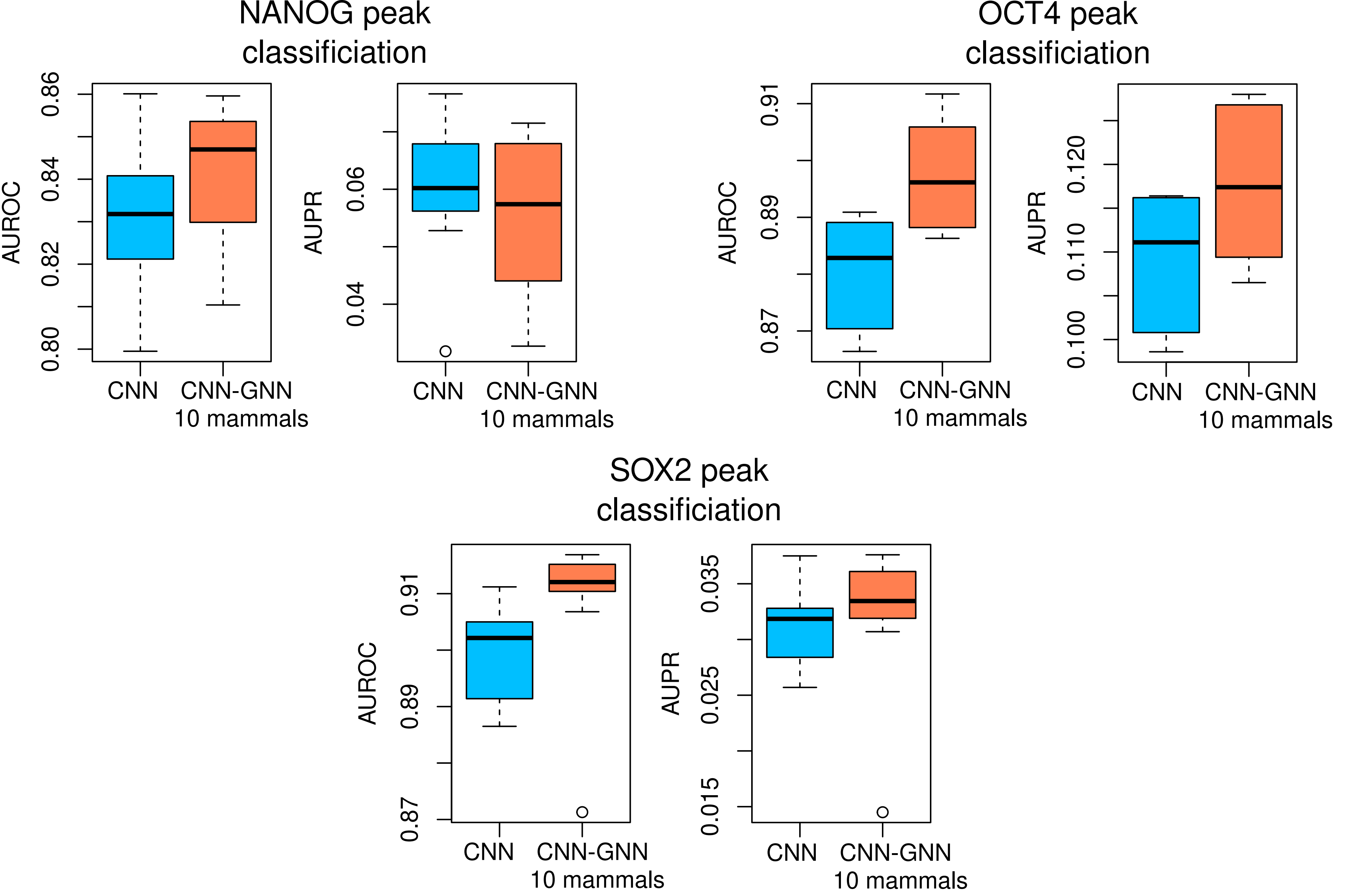

Supp Fig S2: Comparison of prediction performances between the semi-supervised model (here called CNN-GNN) with the baseline model (CNN) where graph convolution was not used. Ten trainings were done for each model to make boxplots. Area under the roc curve (AUROC). Area under the precision recall curve (AUPR).
