# Supplementary material for: Semi-supervised learning improves regulatory sequence prediction with unlabeled sequences
Source: BMC Bioinformatics. 2023 May 5;24:186. doi: 10.1186/s12859-023-05303-2 (PMC10163727; doi:10.1186/s12859-023-05303-2)
Supplement: Supplementary file 3 — Additional file 3: Fig S3. Comparison of prediction performances between different graph neural network convolution layers. A) Comparison in term of area under the roc curve (AUROC). B) Comparison in term of area under the precision recall curve (AUPR). [file 12859_2023_5303_MOESM3_ESM.pdf]

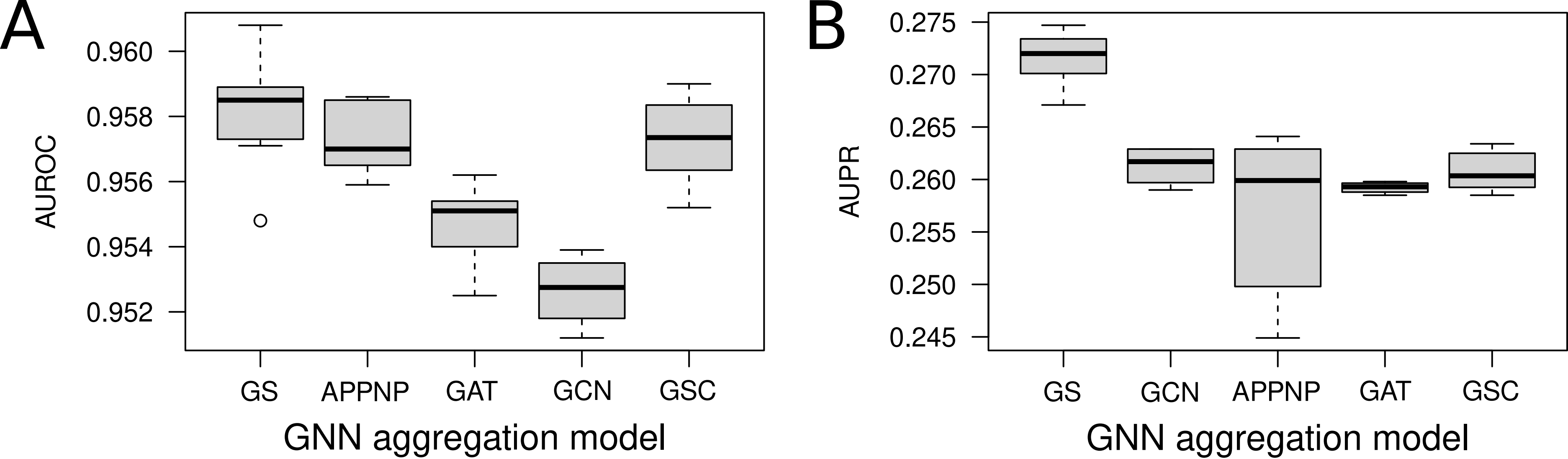

Supp Fig S3: Comparison of prediction performances between different graph neural network convolution layers. A) Comparison in term of area under the roc curve (AUROC). B) Comparison in term of area under the precision recall curve (AUPR).
